# Is the proposed distinction of gaming disorder into a predominantly online vs. offline form meaningful? Empirical evidence from a large German speaking gamer sample

**DOI:** 10.1016/j.abrep.2021.100391

**Published:** 2021-11-01

**Authors:** Christian Montag, Bruno Schivinski, Halley M. Pontes

**Affiliations:** a Department of Molecular Psychology, Institute of Psychology and Education, Ulm University, Ulm, Germany; b School of Media and Communication, RMIT University, VIC 3000 Melbourne, Australia; c Department of Organizational Psychology, Birkbeck, University of London, London, United Kingdom

**Keywords:** Gaming disorder, Internet gaming disorder, Gaming mode, Offline, Online, Gaming device

## Abstract

In the eleventh revision of the International Classification of Diseases (ICD-11), Gaming Disorder (GD) is distinguished between disordered gaming occurring predominantly online, offline, and unspecified. Currently, no study has investigated whether such a distinction is meaningful in diagnosing disordered gaming. Therefore, a large group of gamers with varied tendencies towards disordered gaming was recruited to examine this issue. A large sample (N = 2,768) was recruited and data were collected on disordered gaming, along with information on their preferred gaming mode and device used to play. The present study shows that the distinction between online and offline gaming mode proposed by the WHO is meaningful because online gamers presented with the highest disordered gaming scores followed by mixed gamers (those stating to equally prefer online and offline gaming), and offline gamers. Finally, it was also observed that the type of device for gaming used associated with disordered gaming levels. Specifically, those reporting mostly to use their desktop computer for gaming showed the highest disordered gaming scores. The present study lends empirical support for the consideration of both gaming mode and gaming device in the study of disordered gaming.

## Highlights

• Disordered gaming is distinguished by predominantly online, offline, and unspecific gaming.
• Online gamers showed the highest tendencies towards disordered gaming.
• Gaming via desktop computers was linked with the highest disordered gaming levels.

## 1 Introduction

### 1.1 Background

Video game playing is a popular leisure activity worldwide and an emerging psychosocial phenomenon. However, concerns regarding potential detrimental effects due to excessive and dysregulated gaming engagement leading to disordered gaming have been reported as video game play is a pervasive activity across the entire lifespan (Kircaburun et al., 2020, Pontes, 2018).

As previously suggested (Pontes & Griffiths, 2020), the increased scholarly concerns about the potential addictive effects of video games coupled with the emerging body of empirical evidence supporting the clinical relevance of disordered gaming warranted the inclusion of ‘Internet Gaming Disorder’ (IGD) as a tentative mental health disorder within the fifth revision of the *Diagnostic and Statistical Manual of Mental Disorders* (DSM-5) (American Psychiatric Association, 2013). More recently, disordered gaming has gained further medical recognition as the World Health Organization decided to include Gaming Disorder (GD) in the eleventh revision of the *International Classification of Diseases* (ICD-11) as an official psychiatric condition after extensive controversies and scholarly debates (e.g., Aarseth et al., 2016, Griffiths et al., 2017, Higuchi et al., 2017, Kuss et al., 2017, van Rooij et al., 2018).

In terms of its conceptualization, GD refers to a psychiatric condition in which individuals (i) lose control over gaming, (ii) continue gaming despite the experience of adverse consequences, and (iii) give increasing priority to gaming over previously enjoyed and relevant life interests (World Health Organization, 2020). However, for a GD diagnosis to be present, the WHO suggests that symptoms must occur within a 12-month timeframe and the gaming behavior must lead to significant impairments in everyday life, creating difficulties and functional impairments in several areas of life (e.g., professional, academic, family, romantic) (World Health Organization, 2020).

### 1.2 Structural characteristics in gaming

Although the diagnostic features of GD have been established, little is known about some of its key underlying mechanisms, especially those associated with the way in which video games are developed. It is widely known that different types of video games present with unique addictive potential based on specific design features related to their structural characteristics (Griffiths and Nuyens, 2017, King et al., 2010a, King et al., 2011, Montag et al., 2019a). Structural characteristics within video games refer to those characteristics responsible for inducing play behavior in the first place, or for the inducements related to sustaining play behavior irrespective of the gamer’s psychological, physiological or socioeconomic status (Wood, Griffiths, Chappell, & Davies, 2004). Although different types of video game genres exist (e.g., Multiplayer Online Battle Arena [MOBA], First-Person Shooter [FPS], Massively Multiplayer Online Role-Playing Games [MMORPGs]), these are not typically thought of as structural characteristics per se as structural characteristics comprise the elements and components within video games and not the video games themselves (Griffiths and Nuyens, 2017, Stavropoulos et al., 2020a).

In the context of video game play, several structural characteristics taxonomies have been proposed by early research. The first study to empirically examine the structural characteristics of video games (i.e., Wood et al., 2004) suggested a total of 12 structural characteristics related to sound, graphics, background and setting, duration of game, rate of play, advancement rate, use of humor, control options, game dynamics, winning and losing features, character development, brand assurance, and multiplayer features.

Following this early study, King, Delfabbro, and Griffiths (2010b) identified five broad types of structural features within video games including a total of 24 unique features that are thought to influence playing behaviors uniquely. Accordingly, these five structural features within video games are associated with (i) the social features of video games, (ii) manipulation and control influencing outcomes in video games, (iii) the narrative and identity features allowing players to engage in avatar creation and storytelling, (iv) the reward and punishment aspects of video games determining wins and losses within the game, and (v) the presentation features of video games which impact on auditory and visual experiences (King et al., 2010b).

### 1.3 The role of structural characteristics in GD

In the gambling studies field, structural characteristics have been widely investigated in previous research, with specific taxonomies specifically devised for both online (McCormack & Griffiths, 2013) and offline (Parke & Griffiths, 2007) gambling activities as the means for engaging in the activity (i.e., online or offline) may present with unique addictive potential. Previous research in gambling investigating this issue suggested that online gambling is more likely to contribute to gambling disorder than offline gambling in vulnerable individuals (Griffiths et al., 2009, Wu et al., 2015). Online gambling may heighten people’s risk of getting addicted to gambling (Wu et al., 2015). Internet facilitates gambling activities, availability, and promotes opportunities to gamble, which may lead to greater exposure to the activity and the experience of related harmful outcomes (González-Cabrera et al., 2021).

With regards to the WHO diagnostic framework for gaming disorder (i.e., GD), the WHO distinguishes between GD due to predominantly online gaming activities (6C51.0) and predominantly offline gaming activities (6C51.1) (World Health Organization, 2020). Additionally, a third category referred to as “unspecified” also exists within this diagnostic framework. This third category may characterize individuals who engage in their gaming activities equally online and offline, without a specific focus. Although the addictive potential of other behavioral addictions such as gambling has been investigated in reference to specific structural characteristics such as online and offline (i.e., land-based) gambling, to our knowledge no previous research examined the role of similar structural characteristic features within the WHO diagnostic framework for GD.

It is plausible that the unique design features of video games associated to their structural characteristics might constitute a potential risk factor for GD. One structural characteristic that has not yet been comprehensively investigated relates to multiplayer features, which comprises a specific type of structural characteristic associated with cooperative and interactive gaming behaviors allowing players to socialize with other gamers, form alliances within the game, and to engage in competitive game play (Wood et al., 2004). It is key to investigate the role of structural characteristics in GD as they can be seen as in-built rewards with the potential to elicit compulsive gaming behaviors through a partial reinforcement effect (Griffiths & Nuyens, 2017).

Within the APA diagnostic framework for disordered gaming (i.e., IGD) (American Psychiatric Association, 2013), previous research corroborated the role of multiplayer features as a particularly relevant structural characteristic in disordered gaming (an online feature). In their study, Lemmens and Hendriks (2016) recruited a sample of 2442 gamers to examine the role of online gaming for disordered gaming within the APA diagnostic framework for IGD. Overall, this study found that even though online and offline gaming were both related to IGD, online gaming showed significantly higher associations with IGD than offline gaming (Lemmens & Hendriks, 2016). This finding is unsurprising as the criteria for IGD incorporates the role of the internet (hence the online aspect of GD). Furthermore, disordered gamers were found to spend up to more than four times as much time playing online games (in detail: online role playing games) compared to non-disordered gamers (Lemmens & Hendriks, 2016).

### 1.4 Comorbidities and neuropsychological correlates in GD

Previous empirical research and review studies on GD proposed a wide range of key comorbidities and neuropsychological correlates both predicting and resulting in disordered gaming (see González-Bueso et al., 2018, Karaca et al., 2017, Kuss et al., 2018, Macur and Pontes, 2021, Männikkö et al., 2020, Pontes et al., 2015, Yao et al., 2017). More recently, important factors pertaining specifically to emerging issues linked with variables such as loneliness, attention problems, and depression have been supported in both cross-sectional (Montag et al., 2019b) and longitudinal research (Teng et al., 2021, here depression and anxiety).

A recent study conducted by Moore, Satel and Pontes (in press) investigated whether disordered gaming could be predicted by health and wellbeing factors such as depression, anxiety, loneliness, attention problems, physical health problems, and psychological wellbeing in a sample of young adults and found that about 15% of the variability in GD could be explained by these predictors. In a similar vein, a recent study by Severo et al. (2020) reported that IGD was associated with severe depressive symptoms, poor sleep quality, and increased time spent gaming in a sample of 555 individuals.

At a neuropsychological level, attention deficit has also been established as a correlate of GD. A study by Stavropoulos et al. (2019) found in a cross-cultural sample of Australian and American gamers that those showing higher levels of inattention and hyperactivity symptoms presented with higher levels of disordered gaming. Similarly, recent research (Wang et al., 2020) proposed that the underlying neural mechanism explaining the association between GD and loneliness relates to the effective connection from the left pregenual anterior cingulate cortex to the left laterobasal amygdala as it has been shown to mediate the relationship between the GD and loneliness.

In terms of comorbid depressive symptoms in GD, both longitudinal (e.g., Burleigh et al., 2018, Teng et al., 2021) and cross-sectional (e.g., O’Farrell et al., 2020, Pontes, 2017) research support the links between these two phenomena.

Given that the existing evidence supporting the association between loneliness, attention problems, and depression with disordered gaming have been mostly established under the APA diagnostic framework for IGD, little is known about the extent to which such associations can be replicated under the WHO diagnostic for GD. Therefore, it is key to explore this issue further.

### 1.5 The current study

Despite the initial research by Lemmens and Hendriks (2016), to our knowledge no further research has been conducted to explore the role of structural characteristics (online vs. offline games) in terms of the WHO diagnostic framework for GD. From a scientific standpoint, this is timely as the WHO itself proposed the distinction between online and offline disordered gaming as relevant. Although a substantial amount of video games focusing on the offline gaming experience exists, numerous video games require an internet connection due to their online nature. This is not only true for many Freemium games made for smartphones, but also for classic console games where players can meet with other fellow gamers online to play together. Note that Freemium games refer to video games where the product itself (i.e., the video game) is “free” but potential revenue can be made from the sales of extra in-game features such as avatar customization, new or additional life, increase in health points or energy, additional turns, in-game items, and the like. The sole perspective of Freemium games goes beyond the scope of this study and was not distinguished from online gaming.

With this is in mind, the present study aims to examine the role of internet features within the WHO diagnostic framework for GD to investigate whether online, offline or mixed gamers differ in terms of their GD symptomatology. Given that online games provide a wide range of features and the ability to play with other gamers online, it is expected that GD levels will be higher among online gamers in comparison to offline gamers, while gamers who equally prefer online and offline games being expected to fall in between both the online and offline groups of gamers. In this context, we also mention that tendencies towards attentional problems, depression, and loneliness were assessed because earlier works by Pontes et al., 2019, Montag et al., 2019b demonstrated that these phenotypes are robustly (and positively) associated with higher tendencies towards GD. By inclusion of these measures, the current study will be able to test whether the three gamer groups (i.e., online, offline, mixed) differ in tendencies towards GD, but also with respect to the covarying tendencies related to the aforementioned psychopathological symptoms.

Furthermore, since not all forms of gambling are equally problematic as online gambling presents with differential addictive potential depending on the medium (i.e., device) in which the activity takes place (see Gainsbury, Liu, Russell, & Teichert, 2016), the present study also aimed to investigate whether the device used to play video games would be associated with different patterns of GD.

## 2 Material and methods

### 2.1 Participants

Through the promotion of the team’s German GD self-test-platform via radio, TV, and social media starting in May 2019 to August 2020, a final sample of N = 2,768 (2,356 males and 412 females; mean_age_ = 28.70, SD_age_ = 11.91; Germany = 2126, Luxemburg = 10, Austria = 126, Switzerland = 475, other country = 31; mean-education: 4.36 (SD = 1.89; where 1 = no education to 7 = degree from university) was recruited. A total of 13 participants stated to be professional gamers. Participants were only included when they inserted full and plausible information; were over 11 years (12–17-year-old stated to have parental approval); and mentioned to have played computer games in the last 12 months. The informed consent was provided electronically. Participants with implausible age information were not included in the analyses (e.g., stating to be > 17 years on the informed consent page, but then stated to be minor age). Participants (n = 90; 3.25%), who took the survey in the early stages of the COVID-19 pandemic (between February and August 2020) were not deleted during the data cleaning stage as the exclusion of such participants – given the small overall proportion - would not influence the quality and robustness of the analysis.

Of note, the present study is part of a larger ongoing study conducted by Montag et al. (2019b) where data from 1,429 participants were investigated. This earlier study did not focus on the gaming mode nor on gaming devices used in the context of GD. Note that the only prominent variables not presented in this work stems from a questionnaire assessing gaming motives. Gaming motives have been investigated in detail in this earlier study, and although of interest to the present work, it would deviate from the focus of the present study. Internal consistencies of all questionnaires were adequate (i.e., 0.73 or higher). Participants were incentivized with feedback based on their own answers to some of the measures they filled in. The study was approved by the research team’s University Ethics Committee (N°: 2018/95).

### 2.2 Measures

For the present study, it is of importance that all participants filled in standardized assessment tools examining individual differences in tendencies towards disordered gaming according to both the WHO and APA diagnostic frameworks. Moreover, all participants completed self-reported measures assessing symptoms of loneliness, attention problems, and depression. All participants also provided information on playing predominantly online, offline, or mixed (i.e., equally preferring online and offline games) video games, and if they predominantly used a console, desktop-computer, laptop or a smartphone device for gaming.

#### 2.2.1 Gaming Disorder Test (GDT)

All participants filled in the GDT (Pontes et al., 2021), which consists of four items being answered on a five-point Likert scale (“never” = 1 to “very often” = 5), assessing disordered gaming as per the WHO diagnostic framework. Test scores can range between 4 and 20 points, and higher scores indicate greater tendencies towards GD. Cronbach’s alpha in the present sample was 0.86.

#### 2.2.2 Internet Gaming Disorder Scale–Short-Form (IGDS9-SF)

Participants also filled in the IGDS9-SF by Pontes and Griffiths (2015), which consists of nine items answered within a five-point Likert scale (“never” = 1 to “very often” = 5), assessing disordered gaming as per the APA diagnostic framework. Test scores can range between 9 and 45 points, with elevated scores indicating higher tendencies towards IGD. In the present sample, Cronbach’s alpha was 0.88.

#### 2.2.3 Loneliness, attention problems, and depression

Participants filled in questionnaires on loneliness (UCLA loneliness scale; Russell, 1996), attention problems (attention problems scale; Swing et al., 2010), and the patient health questionnaire without the suicide item (PHQ-8; Kroenke et al., 2001). Higher scores in the scales indicate higher tendencies towards loneliness, attention problems, and/or depressive symptoms.

The three item UCLA loneliness scale was administered with a five-point Likert scale format ranging from “strongly disagree” = 1 to “strongly agree” = 5 (original presented wording in Pontes et al. (2021): “never” = 1 to “often” = 4). In the present sample, its Cronbach’s alpha was excellent (0.87). The attention problem scale, also containing three items, was administered with a five-point Likert scale format ranging from “strongly disagree” = 1 to “strongly agree” = 5. In terms of scoring, both measures can yield scores ranging between 3 and 15 points, with greater scores suggesting higher symptomatology. The attention problem scale presented a Cronbach’s alpha of 0.73. Furthermore, the PHQ-8 questionnaire was administered with a four-point Likert scale format ranging from “not at all” = 1 to “nearly every day” = 4. The PHQ-8 provides total scores ranging from 8 to 32 points, with greater scores suggesting higher levels of depressive symptoms. In the present sample, the PHQ-8 had a Cronbach’s alpha of 0.82. The internal reliability of all the scales used in this study in terms of Cronbach’s alpha were above the recommended literature threshold of 0.70 (Bagozzi and Youjae 1988).

### 2.3 Statistical analyses

We conducted several *t*-tests to investigate the influence of gender on the gaming and psychopathological variables. Correlational analyses were also performed on the main variables of interest to obtain an overview of the main associations in the present sample. For simplicity, we present results from Multivariate Analysis of Covariance (MANCOVAs) with the dependent variables of gaming time (i.e., weekly time spent gaming), GD according to the WHO and APA diagnostic frameworks, as well as attention problems, loneliness, and depression symptoms. While age was inserted as covariate, gender and gaming mode (i.e., online, offline, mixed) were inserted as independent variables. In a second MANCOVA model, the independent variable of gaming mode was exchanged for the gaming device variable (i.e., console vs. desktop computer vs. laptop vs. mobile device such as a smartphone).

Please note that with the large sample size investigated, and with no meaningful differences when using non-parametric tests, we present findings from parametric testing. Given the brevity of this paper, we hint to a full explanation of all measures in the earlier work (Montag et al., 2019b). The following results are presented for the full sample, because no interaction effect with gender was observed. The full data set is available at the Open Science Framework (OSF) (https://osf.io/ntyhr/).

## 3 Results

### 3.1 Descriptive statistics

As presented in Table 1, gender significantly influenced all variables with females presenting lower scores on gaming related variables compared to males (i.e., weekly time spent gaming, GD, and IGD). Statistical significance of the difference of scores across males and females was tested with independent *t*-tests. The reverse picture emerged for attention problems, loneliness, and depressive symptoms variables.

**Table 1:** Descriptive statistics and differences between scores of males and females.

	Total (n = 2,768)	Males (n = 2,356)	Females (n = 412)	*t*-test
Weekly time spent gaming	M = 20.39 (SD = 15.73) Median = 16	M = 20.99 (SD = 15.80) Median = 18	M = 16.97 (SD = 14.85) Median = 14	*t*_(2766)_ = 4.81 *p* < .001
Gaming Disorder	M = 8.69 (SD = 3.62) Median = 8	M = 8.85 (SD = 3.66) Median = 8	M = 7.79 (SD = 3.20) Median = 7	*t*_(614,904)_ = 6.05 *p* < .001
Internet Gaming Disorder	M = 17.44 (SD = 7.03) Median = 15	M = 17.72 (SD = 7.15) Median = 16	M = 15.89 (SD = 6.07) Median = 14	*t*_(628,071)_ = 5.49 *p* < .001
Attention Problems	M = 6.53 (SD = 2.56) Median = 6	M = 6.48 (SD = 2.56) Median = 6	M = 6.81 (SD = 2.56) Median = 7	*t*_(2766)_ = -2.44 *p* < .05
Loneliness	M = 6.22 (SD = 3.04) Median = 6	M = 6.16 (SD = 3.01) Median = 6	M = 6.53 (SD = 3.19) Median = 6	*t*_(__546,660__)_ = -2.20 *p* < .05
Depression	M = 13.78 (SD = 4.35) Median = 13	M = 13.62 (SD = 4.32) Median = 13	M = 14.69 (SD = 4.39) Median = 14	*t*_(__2766)_ = -4.63 *p* < .001

### 3.2 Correlation patterns between the variables of interest

For interested scholars, Pearson correlations are reported between all metric variables of importance (including age). The results indicate that age was inversely and significantly associated with all gaming variables and attention problems. Therefore age, was considered in the subsequent analysis. A summary of the results can be found in Table 2.

**Table 2:** Correlation analysis between metric variables of interest

	Weekly time spent gaming	Gaming Disorder	Internet Gaming Disorder	Attention Problems	Loneliness	Depression	Age
Weekly time spent gaming		*r* = 0.38 *p* < .001	*r* = 0.43 *p* < .001	*r* = 0.10 *p* < .001	*r* = 0.21 *p* < .001	*r* = 0.26 *p* < .001	*r* = −0.14 *p* < .001
Gaming Disorder			*r* = 0.84 *p* < .001	*r* = 0.50 *p* < .001	*r* = 0.41 *p* < .001	*r* = 0.54 *p* < .001	*r* = −0.13 *p* < .001
Internet Gaming Disorder				*r* = 0.47 *p* < .001	*r* = 0.45 *p* < .001	*r* = 0.58 *p* < .001	*r* = −0.15 *p* < .001
Attention Problems					*r* = 0.37 *p* < .001	*r* = 0.53 *p* < .001	*r* = −0.04 *p* < .05
Loneliness						*r* = 0.53 *p* < .001	*r* = 0.03 n.s.
Depression							*r* = 0.002 n.s.

All correlations are presented on two tailed level. Please note that with Spearman correlations effect sizes differ slightly (Pearson correlations are reported here as with the large sample size we prefered to present parametric tests throughout). The Spearman correlations can be found alongside the data via the OSF-link in this paper.

### 3.3 Relationship between gaming mode and gaming device

**Fig. 1:**
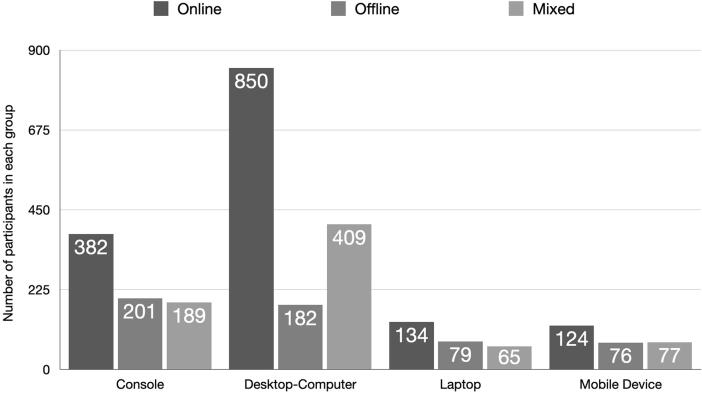
Participants stated which device they usually used to play video games. In all *devices groups* one can see that the most prevailing gaming mode is online. The offline and mixed mode groups are approximately equally large in each device group with the exception of the desktop device, where the *mixed group* is much larger than the offline mode group, but still smaller than the online mode group.

As the present study aimed to investigate the impact of gaming mode (i.e., online, offline, and mixed) and gaming device (i.e., console, desktop computer, laptop, and mobile device) on relevant gaming variables, we checked if these variables could be regarded as independent from each other. To do this, a χ^2^ test was performed, and the results revealed that this was not the case (χ^2^ = 92.22, df = 6, *p* < .001). As can be seen in the Fig. 1, this effect was primarily driven by the group stating to mainly use the desktop computer for gaming. Whereas in all other groups, the device was most often used to play online games, followed by people stating to mainly play offline games, and then the group of gamers playing both online and offline games (see exception mobile device), in the desktop-computer group, a comparably large group of participants stated to use the desktop computer for both online *and* offline gaming.

### 3.4 Testing the variables of gaming mode and gaming device on the dependent variables of interest (including the disordered gaming scales)

As can be seen in Table 3, the gaming mode variable influenced significantly gaming time and disordered gaming variables, but not attention problems, loneliness or depressive symptoms. The pattern that emerged indicated that offline gaming was associated with the lowest weekly time spent gaming and lowest disordered gaming test scores according to both APA and WHO diagnostic frameworks. Moreover, the highest level of disordered gaming and greater weekly time spent gaming was associated with online gaming. Interestingly, the highest effects sizes were observed for the dependent variables “weekly time spent gaming” (about 4% explained variance) followed by the GD scores (each about 2% explained variance).

**Table 3:** Associations between different gaming modes (predominantly online, both online and offline, predominantly offline) and gaming variables, attentional problems, loneliness, and depressive tendencies

	Online (n = 1,490)	Mixed (n = 740)	Offline (n = 538)	Results from MANCOVA
Weekly time spent gaming	M = 23.02 (SD = 16.44)	M = 20.66 (SD = 15.18)	M = 12.73 (SD = 11.38)	*F*_(2,2761)_ = 58.59 *p* < .001 η^2^ = 0.041
Gaming Disorder^a^	M = 9.27 (SD = 3.74)	M = 8.51 (SD = 3.47)	M = 7.32 (SD = 3.04)	*F*_(2,2761)_ = 27.49 *p* < .001 η^2^ = 0.020
Internet Gaming Disorder^b^	M = 18.68 (SD = 7.31)	M = 17.00 (SD = 6.70)	M = 14.63 (SD = 5.66)	*F*_(2,2761)_ = 31.40 *p* < .001 η^2^ = 0.022
Attention Problems^c^	M = 6.64 (SD = 2.61)	M = 6.35 (SD = 2.47)	M = 6.45 (SD = 2.57)	*F*_(2,2761)_ = 0.76 n.s. η^2^ = 0.001
Loneliness^d^	M = 6.15 (SD = 3.08)	M = 6.25 (SD = 2.95)	M = 6.36 (SD = 3.05)	*F*_(2,2761)_ = 0.04 n.s. η^2^ = 0.000
Depression^e^	M = 13.91 (SD = 4.46)	M = 13.69 (SD = 4.38)	M = 13.53 (SD = 3.98)	*F*_(2,2761)_ = 1.32 n.s. η^2^ = 0.001

^a^Test scores can range between 4 and 20 points.

^b^Test scores can range between 9 and 45 points.

^c^Test scores can range between 3 and 15 points.

^d^Test scores can range between 3 and 15 points.

^e^Test scores can range between 8 and 32 points.

**Fig. 2:**
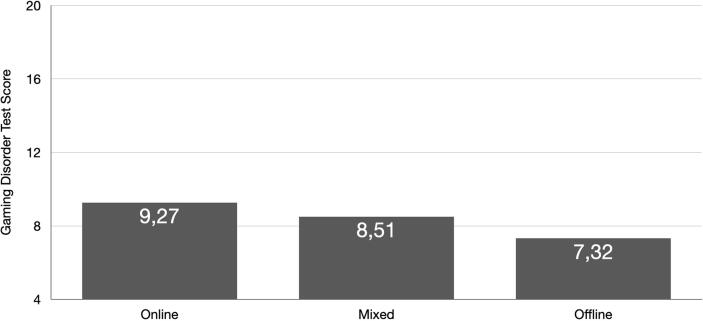
Gaming Disorder Test scores were highest in the group of participants stating to mainly do online games, followed by the group stating to play mostly both types of games (online and offline) and the offline gamers group. Post hoc tests revealed that all groups differed significantly from each other.

In Fig. 2, we depict for better clarity one of the main results from Table 3 showing how gaming mode is associated with GDT scores. Please note that the results are also highly significant if analyses are run, where the influence of gaming mode on GD is calculated, while inserting loneliness, attention problems, and depression as covariates (WHO framework of disordered gaming as dependent variable: F_(2, 2762)_ = 83,41, p < .001, η^2^ = 0.057; APA framework of disordered gaming as dependent variable: F_(2, 2762)_ = 104,87, p < .001, η^2^ = 0.071).

### 3.5 Associations between GD and gaming-related variables

Further analysis revealed that the variable assessing preference for gaming device was robustly associated both with weekly time spent gaming and disordered gaming scores. The results indicated that participants preferring desktop-computers for gaming also reported the highest weekly time spent gaming and greatest levels of disordered gaming (as per the APA and WHO diagnostic frameworks) compared to other gaming devices. A summary of the findings is found in Table 4.

**Table 4:** Associations between different gaming devices and gaming variables, attention problems, loneliness, and depressive tendencies

	Console (n = 772)	Desktop-Computer (n = 1441)	Laptop (n = 278)	Mobile device (n = 277)	Results from MANCOVA
Weekly time spent gaming	M = 17.11 (SD = 13.04)	M = 24.15 (SD = 16.89)	M = 16.50 (SD = 13.77)	M = 13.91 (SD = 12.81)	*F*_(3,2759)_ = 27.86 *p* < .001 η^2^ = 0.029
Gaming Disorder^a^	M = 8.29 (SD = 3.43)	M = 8.99 (SD = 3.68)	M = 8.47 (SD = 3.65)	M = 8.45 (SD = 3.66)	*F*_(3,2759)_ = 4.88 *p* = .002 η^2^ = 0.005
Internet Gaming Disorder^b^	M = 16.98 (SD = 6.87)	M = 17.99 (SD = 7.07)	M = 16.68 (SD = 6.97)	M = 16.65 (SD = 7.12)	*F*_(3,2759)_ = 2.68 *p* < .05 η^2^ = 0.003
Attention Problems^c^	M = 6.48 (SD = 2.63)	M = 6.44 (SD = 2.51)	M = 6.68 (SD = 2.48)	M = 6.97 (SD = 2.70)	*F*_(3,2759)_ = 5.64 *p* < .001 η^2^ = 0.006
Loneliness^d^	M = 5.90 (SD = 2.92)	M = 6.32 (SD = 3.05)	M = 6.59 (SD = 3.27)	M = 6.19 (SD = 3.04)	*F*_(3,2759)_ = 1.91 n.s. η^2^ = 0.002
Depression^e^	M = 13.65 (SD = 4.25)	M = 13.71 (SD = 4.41)	M = 13.99 (SD = 4.43)	M = 14.25 (SD = 4.25)	*F*_(3,2759)_ = 1.30 n.s. η^2^ = 0.001

^a^Test scores can range between 4 and 20 points.

^b^Test scores can range between 9 and 45 points.

^c^Test scores can range between 3 and 15 points.

^d^Test scores can range between 3 and 15 points.

^e^Test scores can range between 8 and 32 points.

No interaction effects between the preferred gaming mode and preferred gaming device variables could be observed on any of the reported variables presented in Table 3, Table 4. Therefore, we refrain from reporting detailed statistical results here. Interested readers can consult the data set used in this study that is available through OSF (https://osf.io/ntyhr/).

## 4 Discussion

The present study aimed to investigate whether gaming mode would be associated with different levels of disordered gaming as such a distinction has been made in the WHO diagnostic framework for GD. According to the results obtained, this distinction is indeed meaningful because individuals playing predominantly online games were associated with the highest levels of disordered gaming in the present study, followed by mixed gamers, and offline gamers. Hence, considering gaming mode helps estimating if a gamer might be more vulnerable for developing disordered gaming. Please note that the explained variance in disordered gaming scores due to gaming mode is in the small area (about 2%), but is slightly larger when testing gaming mode in the context of weekly time spent gaming (about 4%), a variable associated with disordered gaming (GD: *r* = 0.38, hence about 14% shared variance; see Table 2).

The current study did not only investigate gaming mode, but also the preferred gaming device in the context of GD. Interestingly, we first observed that all devices were mainly used for online gaming activities. This is also illustrated by 1,490 participants (53.83%) preferring to play online games, followed by mixed gamers (740; 26.73%), and offline gamers (538; 19.44%). In all gaming device groups, online gamers represented the majority in each group followed by the offline and mixed gamers groups not deviating much in terms of group sizes (the mobile device group consists nearly of the same number of offline and mixed gamers with mixed gamers n = 1 higher). An exception to this rule was the desktop computer group, where the group of mixed gamers was much larger than the offline gamers (mixed gamers are here those stating using the desktop computer for both online and offline gaming).

Finally, we deem it to be highly interesting that those participants stating to play computer games mainly via desktop computers showed both the highest weekly time spent gaming and disordered gaming tendencies. This needs to be discussed in the future also against the background of Freemium games (Montag, Lachmann, et al., 2019a), which are known to have built-in gaming elements likely to prolong online play time. Unfortunately, this is an understudied research area with much knowledge likely existing in the hands of the gaming industry, who can facilitate additional research able to tackle this issue (Griffiths & Pontes, 2020), but not independent researchers working isolated (see also a comparable situation with social media platforms; Montag et al., 2021). In general, it is plausible that online gaming is associated with highest disordered gaming levels (see also Lemmens & Hendriks, 2016), as online gaming provides more exciting gaming elements (e.g., multiplayer features) compared to offline games.

With regards to the comorbidities and correlates investigated in the present study, we found evidence supporting a positive association between disordered gaming with loneliness, attention problems, and depression. More specifically, disordered gaming as assessed under the WHO and APA diagnostic frameworks both exhibited a highly comparable association pattern (Montag et al., 2019b). The findings obtained echo the findings of previous research reporting a positive association between IGD with loneliness (e.g., Montag et al., 2019b, Rogier et al., 2021, Wang, 2021), attention problems (e.g., Chen et al., 2021, Montag et al., 2019b, Yen et al., 2017), and depression (e.g., Evren et al., 2019, Jeong et al., 2019, Montag et al., 2019b). Despite corroborating previous findings, and the expected association between disordered online gaming and increased tendencies towards psychopathology, our study findings warrant further scrutiny of the inter-relationships between GD and other comorbidities that have been proposed in the context of IGD, particularly in the context of clinical samples diagnosed with GD.

Although this study did not observe differences in behaviours across the gender of gamers  (here we touch upon interaction effects with our main research questions; well-known main effects of gender on several variables are presented in Table 1), our findings evidenced that the age of gamers was significantly associated with all gaming variables and investigated attention problems. The literature indicates that emerging adulthood is a critical point in the prevention of psychiatric disorders and future chronic disease (Micallef et al., 2021). ﻿This is of great concern as young gamers, if unassisted, may spend long hours playing video games, which may trigger disordered gaming symptoms such as withdrawal (Coyne et al., 2015) and loss of control in the activity (Antons et al., 2020) - both core drivers of IGD as evidenced in the literature (Pontes et al., 2019).

Despite its contributions, the present study comes with limitations. As a consequence, the findings presented and discussed here should be interpreted with caution. Although robust statistical methods and valid psychometric tests were administered, the present study based on self-reported data from individuals. Such a limitation is of importance to be mentioned in the study of problematic technology use behaviours (Rozgonjuk et al., 2021), since self-reports are not perfect instruments to check on mental status and disordered behaviours. We, therefore, recommend researchers to further validate the presented findings using different methodologies (e.g., structured interviews) and other samples (e.g., using clinical samples).

A further limitation needs to be mentioned: although large, the investigated sample was not representative of the German speaking (gamer) population. Moreover, as often observed in the gaming literature, only a few female gamers could be recruited for this study, and as such, future research should more strongly aim to investigate population of female gamers. We also recommend further research to extend the findings presented in this study by investigating specific structural characteristics and differences between gaming platforms and gameplay. For instance, researchers could investigate the differences between analog and digital games, virtual simulators, and avatars (Stavropoulos et al., 2020b) and to which extent they relate to the presented findings. This is of major interest as gaming structural characteristics relate to the immersive and reality-shifting nature of modern gaming.

Furthermore, the present study is cross-sectional and of correlational nature, forbidding causal inferences between variables. Hence what we presented in this study are associations between variables.

## 5 Conclusions

Despite the aforementioned potential limitations, the authors are optimistic, that the present insights are of value, showing that the distinction between online and offline gaming is meaningful and that even gaming devices are of interest when considering the assessment of disordered gaming under the APA and WHO diagnostic frameworks.

## Contributions

CM designed the present study. CM and HP wrote the manuscript. CM ran the statistical analysis. BS critically revised the paper.

## Funding

None.

## Declaration of Competing Interest

The authors declare that they have no known competing financial interests or personal relationships that could have appeared to influence the work reported in this paper.
